# A national research agenda for pre-hospital emergency medical services in the Netherlands: a Delphi-study

**DOI:** 10.1186/s13049-015-0195-y

**Published:** 2016-01-08

**Authors:** Irene van de Glind, Sivera Berben, Fon Zeegers, Henk Poppen, Margreet Hoogeveen, Ina Bolt, Pierre van Grunsven, Lilian Vloet

**Affiliations:** HAN University of Applied Sciences, Department of Emergency and Critical Care, PO Box 6960, 6503 GL Nijmegen, The Netherlands; Radboud university medical center, Radboud Institute for Health Sciences, IQ healthcare, P.O. Box 9101 (114), 6500 HB Nijmegen, The Netherlands; Radboud university medical center, Regional Emergency Healthcare Network Nijmegen, PO BOX 9101 (911), 6500 HB Nijmegen, The Netherlands; HAN University of Applied Sciences, Institute of Nursing Studies, PO BOX 6960, 6503 GL Nijmegen, The Netherlands; Dutch National Sector Organisation for Ambulance Care (Ambulancezorg Nederland, AZN), PO BOX 489, 8000 AL Zwolle, The Netherlands; Dutch Nurses Association, department of Ambulance Care (V&VN, ambulance care), PO Box 8212, 3503 RE Utrecht, The Netherlands; Regional Emergency Medical Service Veiligheidsregio Gelderland-Zuid, PO BOX 1120, 6501 BC Nijmegen, The Netherlands; Nursing Advisory Board and scientific office, Canisius Wilhelmina Hospital, PO BOX 9015, 6500 GS Nijmegen, The Netherlands

**Keywords:** Ambulance, Emergency Medical Services, Evidence based practice, Research priorities, Delphi study

## Abstract

**Background:**

In pre-hospital Emergency Medical Services (EMS) more research is needed to direct and underpin care delivery and inform policy. To target future research efforts, this study aimed to determine future research priorities with representatives of the EMS field.

**Methods:**

A four-round online Delphi survey was used to discuss different viewpoints and reach consensus on research priorities. A multidisciplinary panel of experts was recruited in the field of pre-hospital EMS and adjoining (scientific) professional organisations (*n* = 62). 48 research topics were presented in Delphi I, and the panel was asked to rate their importance on a 5-point scale. In Delphi II and III the panel selected their priority research topics, and arguments why and suggestions for research questions were collected and reported back. In Delphi IV appropriateness of the remaining topics and agreement within the expert panel was taken into account to make up the final list of research priorities.

**Results:**

The response on the Delphi-survey was high: 95 % (*n* = 59; Delphi I); 97 % (*n* = 60, Delphi II); 94 % (*n* = 58, Delphi III); 97 % (*n* = 60, Delphi IV). The panel reduced the number of research topics from 48 topics in Delphi I to 12 topics in Delphi III. A variety of arguments and suggestions for research questions were collected, giving insight in reasons why research on these topics in the near future is needed.

Delphi IV showed an adequate level of agreement with respect to the 12 presented research topics. The following 9 topics were rated as appropriate for the national pre-hospital EMS research agenda: Non-conveyance to the hospital (ranked highest); Performance measures for quality of care; Hand over/registration/exchange of patient data; Care and task substitution; Triage; Assessment of acute neurologic signs & symptoms; Protocols and protocol adherence; Immobilisation; and Open/secure airway.

**Discussions:**

The research priorities identified in our study resemble those in other studies. However, the topic 'non-conveyance to the hospital' was determined as a priority in this study but not in other studies.

**Conclusions:**

The national pre-hospital EMS research agenda can focus future research efforts to improve the evidence base and clinical practice of pre-hospital emergency medical services. Dissemination and implementation of the research agenda deserves careful attention.

## Background

Research is essential to direct and underpin the delivery of care, and to contribute to better patient outcomes [1, 2]. In pre-hospital Emergency Medical Services (EMS) the principles of evidence-based practice are also supported. However, compared to other healthcare settings, conducting research in this field is difficult partly because research capacity and expertise are limited. Furthermore, a lack of high quality EMS research is related to the fact that randomized controlled trials are difficult to conduct in this setting [3–5]. Therefore, research in EMS is currently often small scale [6–8] and findings are frequently of limited applicability in the specific context of pre-hospital care [6, 7]. Many national and international researchers, professionals, and stakeholders advocate and encourage an increase of high quality research in the pre-hospital emergency services setting [2–7, 9].

Various promising developments in the (Dutch) pre-hospital EMS arena are contributing to increase the evidence-base and the quality of emergency care delivery, such as projects to develop performance measures, the implementation of national guidelines, and the implementation of patient safety programs [10–14]. Moreover, research capacity and research expertise in ambulance care services is increasing due to more paramedics gaining postgraduate qualifications [4]. In the Netherlands, a recent survey identified that 64 unique research projects were conducted in ambulance care organisations in the period 2012–2014 [15]. The majority of these identified research projects focused on cardiac topics. Moreover, only one third of the projects were coordinated by ambulance care organisations themselves. This indicates that mostly others related to the EMS field took the initiative to conduct research on certain topics [15].

In view of these developments, the Dutch National Sector Organisation for Ambulance Care (Ambulancezorg Nederland) supports the development of a national research agenda for pre-hospital EMS to further advance and focus research efforts.

From other healthcare professions and domains we learn that a research agenda can be very helpful to target research efforts and to provide a framework for future investments [16–20]. Canada, the USA, Australia, the UK and Ireland already set an agenda to prioritize research efforts in the field of EMS [21–23]. However, since pre-hospital emergency care is very diversely organised, research priorities in Canada or Australia are not necessarily one-to-one transferrable to the Netherlands. The Dutch EMS context is characterised by regional EMS ambulance organisations who employ ambulance nurses. These are all registered nurses (RN) who have a mandatory bachelor education with ample experience in intensive care or emergency care. Additionally, they are qualified as emergency medical technician level four.

To produce research that informs and directs evidence based practice and policy in the Netherlands, priorities are preferably related to the (country) specific context and care system. In order to develop a research agenda that can count on support and commitment from practitioners, research priorities are preferably determined by representatives from all the disciplines working in the field of pre-hospital EMS and related professional organisations (e.g. medical specialists; The Dutch College of General Practitioners) and interest groups (e.g. the Ministry of Health, Welfare and Sports; the Advisory board for Ambulance EMS) in the Netherlands. The aim of this study was therefore to develop a national pre-hospital EMS research agenda, with representatives of the EMS field, to determine future research priorities in the Netherlands.

## Methods

### Design

A four-round Delphi survey technique was used as the study design. This group facilitation technique aims to obtain consensus on the opinions of ‘experts’ through a series of structured questionnaires [24]. We designed the Delphi survey to collect arguments, discuss different viewpoints and to reach consensus on research priorities in the field of pre-hospital EMS. Using this design, the information flow could be structured and the input of the participants could be reported back effectively. Moreover all participants remain anonymous during the study. This prevents that authority, status, personality, or reputation of group members can influence (and bias) the process and the outcome.

To increase validity all Delphi surveys were developed and pretested by an EMS research-working group, with representatives of the national EMS association for nurses, EMS physicians and the Dutch National Sector Organisation for Ambulance Care. The list of Hasson et al. on Delphi survey techniques was used as quality check for this article [24]. The study was assessed by the Medical Ethics Committee of the district Arnhem – Nijmegen in the Netherlands. They concluded that, according to Dutch Law, this study was deemed exempt from their approval.

### Delphi panel

Purposeful sampling was used to recruit different pre-hospital EMS professionals in the Delphi panel: medical managers of ambulance care organisations, ambulance nurses, ambulance drivers, ambulance care dispatchers, physician assistants, nurse specialists, nurse educators, and researchers. Other experts involved in the panel were managers and policy advisors from ambulance care services and representatives of organisations closely related to the field of EMS, such as national professional associations (e.g. emergency physicians, emergency nurses, trauma surgeons, and anaesthesiologists), see Table 1. The inclusion of pre-hospital EMS experts in the panel was organized by the Dutch National Sector Organisation for Ambulance Care, taking into account the different geographical regions and different professions working in the field. Other groups closely related to pre-hospital EMS were recruited by sending a letter to the board of the national associations of (medical) professions or other institutions. We asked them to assign a delegate on behalf of their association to participate in the study. This strategy resulted in a multidisciplinary panel of experts (*n* = 62).

**Table 1:** Characteristics of the Delphi panel

Professionals directly involved in pre-hospital EMS		Professional and interest groups related to EMS	
Ambulance care dispatcher	2	The Netherlands Society of Anesthesiology	1
Ambulance driver	2	The Netherlands Society of Cardiology	1
Ambulance nurse	12	The Netherlands Society of Neurology	1
Physician assistant/clinical nurse specialist	7	The Netherlands Society for the Surgery of Trauma	1
(Nurse) Researcher	3	The Netherlands Society of Obstetrics and Gynecology	1
Education coordinator/teacher/educator	3	The Netherlands Society of Internal Medicine	1
Medical manager ambulance care	8	The Dutch College of General Practitioners (NHG)	1
Health policy advisor	3	The Netherlands Society of Emergency Medicine	1
Board of directors/middle-management	4	The Netherlands Society of Emergency Nursing	1
		Advisory board for Ambulance EMS	1
		Ministry of Health, Welfare and Sports	1
		National Emergency Healthcare Network/National Helicopter EMS steering board	1
		Researchers and health policy advisors	6
Subtotal	44		18
Total			62

### Data collection

The Delphi study consisted of four consultation rounds using electronic surveys, running from May 2013 until June 2014. In each Delphi round we provided the panel with feedback on the results of the previous consultation.

A previously identified national framework of pre-hospital EMS research topics was used as starting point of this Delphi study [van de Glind et al., submitted]. The framework consists of 11 categories and 48 research topics (Table 2), describing medical care components such as airway management, and organisational themes such as developing quality indicators to measure quality of care.

**Table 2:** Pre-hospital EMS research topics

Airway, Breathing and Pulmonology
– Open/secure airway (e.g. intubation)
– Mechanical ventilation (devices)
– Auscultation
– Oxygen supply
– Pharmaceutical intervention in COPD
– CO2
– Hyperventilation
Circulation and Cardiology
– Shock therapy
– Resuscitation & devices, and therapeutically hypothermia treatment
– Assessment of acute cardiac signs & symptoms
– Diagnostics/ECG for acute cardiac signs & symptoms
– ACS management
– Sepsis
Disability, Exposure and Neurology
– Pain management
– Assessment of acute neurologic signs & symptoms
– Intoxication (alcohol/drugs)
– Neurological exam (Glasgow Coma Scale)
– (Unintentional) hypothermia
Traumatology
– Immobilisation
– Consult Helicopter Emergency Medical Service
– Pain management in trauma
Internal medicine
– Acute gastro-intestinal complaints
– Diabetes
Gynaecology/Obstetrics
– Childbirth: CTG, care management for young born & mother
– Maternal haemorrhage
Psychiatry
– Emergency psychiatric care
Organisation of care
– Care and task substitution (e.g. MANP & PA)
– Cost-effectiveness
– e-Health
– Non-conveyance to hospital
– First responders (police, fireman, citizens)
– Rapid responders
– Availability of ambulance EMS
Collaboration in the chain of emergency care
– Registration
– Hand over/ registration / exchange of patient data
– Feedback/evaluation
– Safety of employees
Quality of Care
– Performance measures for quality of care
– Patient Safety
– Triage
– Protocols and protocol adherence
– Stay and play versus scoop and run
– Patient perspective
Education
– Professional behaviour
– Competences (knowledge, skills and attitude)
– Education programs (e.g.. simulation)
– e-Learning
– Tests and exams (e.g. peer assessment)

The four Delphi rounds were designed to reach consensus about the pre-hospital EMS research priorities. First, the number of topics was reduced from 48 to 25 by selecting the highest ranked 25 topics in Delphi I. Then, in Delphi II, reasons why these topics are considered important were collected, and the number of topics was further reduced from 25 to 10. All collected arguments and reasons were reported back, and the panel was asked to select their top-3 of research topics in Delphi III. Finally, Delphi IV was designed to reach consensus about the remaining 12 research topics. Below, the design of the four Delphi rounds is described in detail. The analysis of the data collected in the Delphi rounds will be described in the next paragraph.

All 48 research topics were included in Delphi I, where we asked the panel to rate the importance of each topic on a 5-point Likert scale from 1 (not important) to 5 (very important). As collecting arguments on the initial research topics (*n* = 48) would be too extensive for an adequate discussion in the expert panel, we first aimed to reduce the amount of topics.

In Delphi II the 25 top priority research topics were displayed to the panel, and the experts were asked to select a top 10 of topics (by answering yes/no per research topic), and to argue their choice. We aimed to collect a full array of arguments (pros and cons) for every research topic. Also, suggestions for research questions regarding the chosen topics were collected. Additionally, participants could reselect one research topic that was discarded after round I.

In Delphi III the panel was asked to select a top 3 of research topics (by answering yes/no per research topic) taking into consideration all arguments given in the previous round. Experts could give additional arguments why they selected certain research topics for the national pre-hospital EMS research agenda. Furthermore, new research questions regarding the research topics were collected. Additionally, participants had the opportunity to reselect one research topic previously dropped off the list.

In the final Delphi IV, the panel was asked to rate the importance of the remaining 12 research topics on a 9-point Likert scale from 1 (not important) to 9 (very important). The 9-point Likert scale was chosen to be able to calculate and test the level of importance of the topics as well as level of agreement between the participants, following the RAND/UCLA appropriateness method [25].

### Analysis

In Delphi I, all 48 potential research topics were ranked, based on a calculated priority score (the number of positive ratings (score 4 and 5) minus the number of negative ratings (score 1 and 2)). The 25 most important research topics (based on the calculated priority score) were presented in a list as input for Delphi II.

In Delphi II and III, the remaining 25 research topics were ranked based on a calculated score (the number of positive ratings (yes) minus the number of negative ratings (no)). Arguments to select (pro) or reject (con) a topic for the national research agenda were qualitatively analysed. Two researchers (IVDG, FZ) independently read all answers given by the panel, identified and summarized unique arguments. Then, all arguments were checked and discussed by the two researchers and in case of disagreement a third (senior) researcher was consulted (SB) to achieve consensus.

In Delphi IV we used the RAND/UCLA appropriateness method to determine the topics of the research agenda, following the classification of appropriateness and agreement. Median scores were calculated for each research topic. Scores between 7 and 9 were defined as appropriate, 4 to 6 as somewhat appropriate, and 1 to 3 as not appropriate. To determine agreement among participants of the Delphi panel on these topics, the disagreement index was calculated for each topic [25]. A disagreement index of less than 1 was regarded as adequate, according to the recommendations of Fitch et al. for defining agreement in Delphi surveys [25]. Finally, each research topic was placed into a category, based on the median score rating the importance of the topic together with the disagreement index regarding this topic. We used three categories [24]: (1) the topic is appropriate (median of 7–9) and there is consensus within the panel; (2) the topic is possibly appropriate (median 7–9), however without consensus or the topic is somewhat appropriate (median 4–6) with or without consensus in the panel; and, (3) the topic is not appropriate (median 1–3) (with or without consensus in the panel). All topics within category 1 (appropriate and consensus in the panel) were added to the final list of pre-hospital EMS research priorities.

## Results

The response on the Delphi-survey was high: 95 % (*n* = 59; Delphi I); 97 % (*n* = 60, Delphi II); 94 % (*n* = 58, Delphi III); 97 % (*n* = 60, Delphi IV). Non-response was random, and reasons for non-response were: lack of time, being abroad, or quit working for the EMS organisation at the time of one of the rounds.

The panel selected 25 topics in Delphi I, and further reduced these to 12 topics for the EMS research agenda in Delphi III. Table 3 presents the ranking of topics in Delphi II and III. The topic ‘non-conveyance’ was the topic most frequently selected in the first three rounds.

**Table 3:** Ranking of research topics Delphi II, III

Ranking Delphi III	Ranking Delphi II	
1	1	Non-conveyance to hospital
2	5	Hand over/registration/exchange of patient data
3	8	Care and task substitution (MANP & PA)
4	7	Performance measures for quality of care
5	2	Pain management
6	9	Resuscitation & devices
7	4	Immobilisation
8	6	Sepsis
9	21	Triage
10a^a^	10	Assessment of acute neurologic signs & symptoms
10b^a^	15	Protocols and protocol adherence
11	3	Open/secure airway (e.g. intubation)

^a^shared ranking

Arguments given in round II and III to select topics for the national pre-hospital EMS research agenda were very diverse. In Table 4 some examples of arguments are illustrated.

**Table 4:** Examples of arguments why to select a topic for the research agenda (Delphi II, III)

*Arguments with respect to Non-conveyance*
Non-conveyance occurs frequently;
There are several (potential) risks related non-conveyance;
Legal issues and risks for the paramedics involved.
*Arguments with respect to ‘Hand over/registration/exchange of patient data’*
A nationwide registration method is currently lacking;
Patient data are essential to monitor and ensure a good quality treatment in the chain of emergency care (General Practitioner, Ambulance EMS, Helicopter EMS (HEMS) and Emergency Department ED);
Unstructured handovers are seen as an important risk factor for adverse events and unnecessary delay in the emergency health care process.

Next to arguments, the panel proposed several suggestions of research questions (in Delphi II and III) to be addressed in future studies (see Table 5). For ‘Non-conveyance to hospital’, the panel reported various research questions to assess the incidence of non-conveyance; to investigate characteristics of this specific population who was not transported to the hospital (after the 911 call); to investigate adverse events and risks related to the decision of non-transport; and, to identify determinants that influence the decision of the paramedic not to transport the patient to the emergency department (ED).

**Table 5:** Suggestions of research questions to be addressed

Research topic	Suggestions of research questions to be addressed (collected in Delphi round II, III)
Non-conveyance	– How many times does non-transport occur? – What are patient characteristics of this group? – Was it the right decision not to transport the patient to the hospital afterwards (morbidity/mortality rates)?
Performance measures for quality of care	– What is the quality of ambulance care in the Netherlands – and is there regional variation?
Hand over/registration/exchange of patient data	– What patient information should be registered uniformly nationwide? – What is the most effective way to handover patient data and information from ambulance to hospital?
Care and task substitution (MANP & PA)	– What tasks can a MANP and PA carry out in the EMS field? – What are the effects of care/task substitution on quality of care and costs?
Triage	– What are the effects of different triage tools (such as NTS and ProQa)?
Assessment of acute neurologic signs & symptoms	– Can ambulance nurses effectively assess acute neurologic signs and symptoms in order to start thrombolytic-treatment earlier?
Protocols and protocol adherence	– To what extent do EMS professionals adhere to protocols? – When do professionals deviate from protocol, and why?
Immobilisation	– When and how to immobilise patients in pre-hospital setting? – How to determine which patients should be immobilised?
Open/secure airway (e.g. intubation)	– What is the effectiveness of different devices for intubation? – What is the most effective device for open/secure airway in children?

The results of Delphi IV are presented in Table 6. The disagreement index, the measure for consensus, was lower than 1 for all topics. This indicated that the panel had an adequate level of agreement with respect to all topics. In Delphi IV, nine out of twelve topics were rated as appropriate for the national pre-hospital EMS research agenda because these topics were rated a median of 7 or higher. Median scores were all between 6 and 9, indicating that no single topic was regarded as not appropriate. The topic ‘non-conveyance’ received the highest rank. Two research topics had decimal medians (6.5 for the topics Immobilisation and Open/secure airway), due to an even number of people in the panel. In order to favour the expert opinion, these topics were included in the appropriateness category and thus added to the research agenda. Three research topics had a median score of 6 and were therefore considered somewhat appropriate (category 2), these topics were not added to the national pre-hospital EMS research agenda.

**Table 6:** Median and Disagreement Index of the research topics Delphi IV (*n* = 60)

	Median (1–9)	Disagreement Index (> 1 disagreement)	Category^a^
Non-conveyance	9	0.13	1
Performance measures for quality of care	7.5	0.16	1
Hand over/registration/exchange of patient data	7	0.16	1
Care and task substitution (MANP & PA)	7	0.37	1
Triage	7	0.37	1
Assessment of acute neurologic signs & symptoms	7	0.56	1
Protocols and protocol adherence	7	0.65	1
Immobilisation	6.5	0.52	1
Open/secure airway (e.g. intubation)	6.5	0.52	1
*Pain management*	*6*	*0.52*	*2*
*Resuscitation & devices*	*6*	*0.56*	*2*
*Sepsis*	*6*	*0.52*	*2*

^a^Category 1: the topic is appropriate (median of 7–9) and with consensus (disagreement index < 1); Category 2: the topic is appropriate (median 7–9), however without consensus, or the topic is somewhat appropriate (median 4–6) with or without consensus; and Category 3: the topic is not appropriate (median 1–3) with or without consensus in the panel. All topics within category 1 were added to the ambulance EMS research agenda

## Discussion

In this study a national pre-hospital EMS research agenda was developed with a multidisciplinary group working in the field of pre-hospital EMS in the Netherlands. In a four-round Delphi study 48 research topics were prioritized, of which nine were considered appropriate and met criteria for adequate levels of agreement. Additionally, a variety of arguments and suggestions for research questions were collected, giving insight in reasons why research on these topics in the near future is needed. The development of a research agenda is a valuable first step to further increase the evidence-base in pre-hospital emergency care delivery, and produces research that informs and directs practice and policy.

Other countries have launched similar initiatives to identify EMS research priorities [3–5, 21, 23, 26–29]. All these studies clearly promote and encourage more scientific research in the pre-hospital emergency care setting. Comparing the research priorities of this study with topics of other studies gives insight in similarities and differences between the research topics.

Our study demonstrates a high level of interest in the topic *Non-conveyance.* In both Canada and the UK, non-conveyance was mentioned as research topic, but was no priority [21, 28]. The topic was formulated as *‘Safety, costs and benefits of alternatives to conveyance to hospital’* in the UK, and *‘Destination decisions, non-transport and alternatives to referrals by EMS providers’* in Canada. This might indicate that pre-hospital services across countries feel the pressure to deal with scarce resources and an increase in calls. One possible explanation why non-conveyance was ranked highest in the Netherlands but not in other countries is the relative autonomy of ambulance nurses in The Netherlands. Another argument could be that access to the emergency health care system differs between countries. In the Netherlands, a patient can receive urgent emergency care from different organisations : the general practitioner (GP) during office hours; an out of hours primary care cooperative; the emergency department (ED), and furthermore a 911-call provides access to emergency care provision of an ambulance [30]. Due to a shift in the accessibility of primary care (long waiting times), people might more often make a 911-call. While probably they could have called the out of hours primary care service. According to the expert panel of this study, non-transport occurs frequently in current practice, however the exact rates are yet unknown. Other research questions related to non-conveyance were gaining insight into characteristics, determinants, and risks and (cost saving) benefits of the decision not to transport the patient to the hospital.

The other research priorities determined in our study resembled those in other countries. The topic ‘Performance measures for quality of care’ was reported in Canada, the UK and the USA [4, 21, 28, 31]. As pointed out by Snooks et al., response time is often used as the single performance measure, while research has shown that this measure is not sufficiently adequate to measure quality of ambulance services [28]. The topic ‘Hand over/registration/exchange of patient data’ was reported as priority in Canada, Australia and the UK [21, 28, 29]. Remarkably, this topic was also identified as barrier for conducting research [27]. Electronic standardised patient care reports and reliable databases are needed to conduct high quality research, but these are often not available. Furthermore, the topic ‘care and task substitution’ was also reported in other studies, such as: the effects of regionalisation of care for specific conditions [21]; the role of the paramedics in various health settings [21, 29]; the role and benefit of advanced practitioners (physician manned ambulances) [3, 26]; and the relationship between skill level and outcome [3, 29]. Other similar priority topics concerned specific interventions for which the evidence in the pre-hospital field is scarce. Examples of such knowledge gaps were given with respect to airway management, immobilisation, and resuscitation and devices [3, 26, 28, 29]. Furthermore, the topics triage and protocol-adherence were also identified as priorities in other studies [3, 21, 26, 27].

There were a number of research priorities in other countries not identified in our study. First, one study reported the need for evidence on introducing pre-hospital ultra sound [26], research with respect to ergonomics, workforce, lifting and equipment [21, 29]. Furthermore, Cone et al. recommended more research how the field adopts new scientific evidence, and the role of (commercial) advertising in this process– in particular when evidence is scarce or conflicting [3]. Next to research priorities, previous studies identified barriers for conducting scientific research in the field of emergency medicine [27–29, 32]. Several recommendations to solve these barriers were suggested, such as improving training opportunities for EMS researchers, stimulating increases in available funding sources, and facilitation of protected time for staff to conduct research.

Apparently, many countries feel the need to develop a research agenda and to encourage conducting scientific research in the field of EMS. Despite some differences between countries and systems, we identified many similarities in future research topics. This could stimulate researchers to collaborate internationally with respect to high priority research questions, and thereby increase the evidence for pre-hospital patient care delivery.

The Dutch national pre-hospital EMS research agenda contains broadly defined research topics. To take this agenda further, we need to translate these *potential* research priorities to *actual* research questions. A literature study should be conducted to summarize the evidence and existing body of literature on the research priorities of the national agenda. Dissemination and implementation of the national research agenda deserves careful attention, taking into account barriers and facilitators influencing these processes. From the initiatives in other countries, we learn that the pre-hospital EMS field needs to be facilitated to implement the research agenda [3, 21–23, 27]. Research skills need to be introduced into the working environment and the education system. A research community needs to be created where knowledge translation and application is facilitated. Senior management should encourage and support research. And, additional funding of research in the pre-hospital emergency setting is necessary.

This is the first study in the Netherlands that developed a national pre-hospital EMS research agenda. A strength of this study is the high response of the multidisciplinary expert panel in all rounds of the Delphi study. This indicates enthusiasm, commitment and broad support for the national research agenda. Furthermore, the rich qualitative data from this study is very useful to understand why topics are research priorities, and what specific research questions are suggested to focus future research on. This combination of quantitative and qualitative data is valuable, and we recommend other researchers to also collect qualitative data when they want to develop a research agenda.

Some limitations of this study should also be discussed. In Delphi IV we used the RAND/UCLA appropriateness method [25], taking into account agreement within the panel. In hindsight, it would have been better to use the same criteria for selecting research topics in each Delphi round. But, considering the high level of agreement on the topics in the final Delphi round, we assume that results would have been the same anyway. Another limitation of the study is that we did not succeed in including the patient perspective in the expert panel. Although a patient representative was invited to take part in the expert panel, the invitation was not (or could not be) accepted. The patient perspective should be included in the implementation of the research agenda, for instance when formulating actual research questions, commissioning a call for research or decisions on funding research proposals.

## Conclusions

A multidisciplinary expert panel identified pre-hospital EMS research priorities in the Netherlands. The research topics include: Non-conveyance; Performance measures for quality of care; Hand over/registration/exchange of patient data; Care and task substitution; Triage; Assessment of acute neurologic signs & symptoms; Protocols and protocol adherence; Immobilisation; and Open/secure airway. These topics provide a focus for future research efforts to improve the evidence base and clinical practice of pre-hospital emergency medical services. Dissemination and implementation of this national EMS research agenda deserves careful attention.
